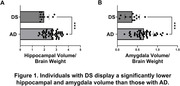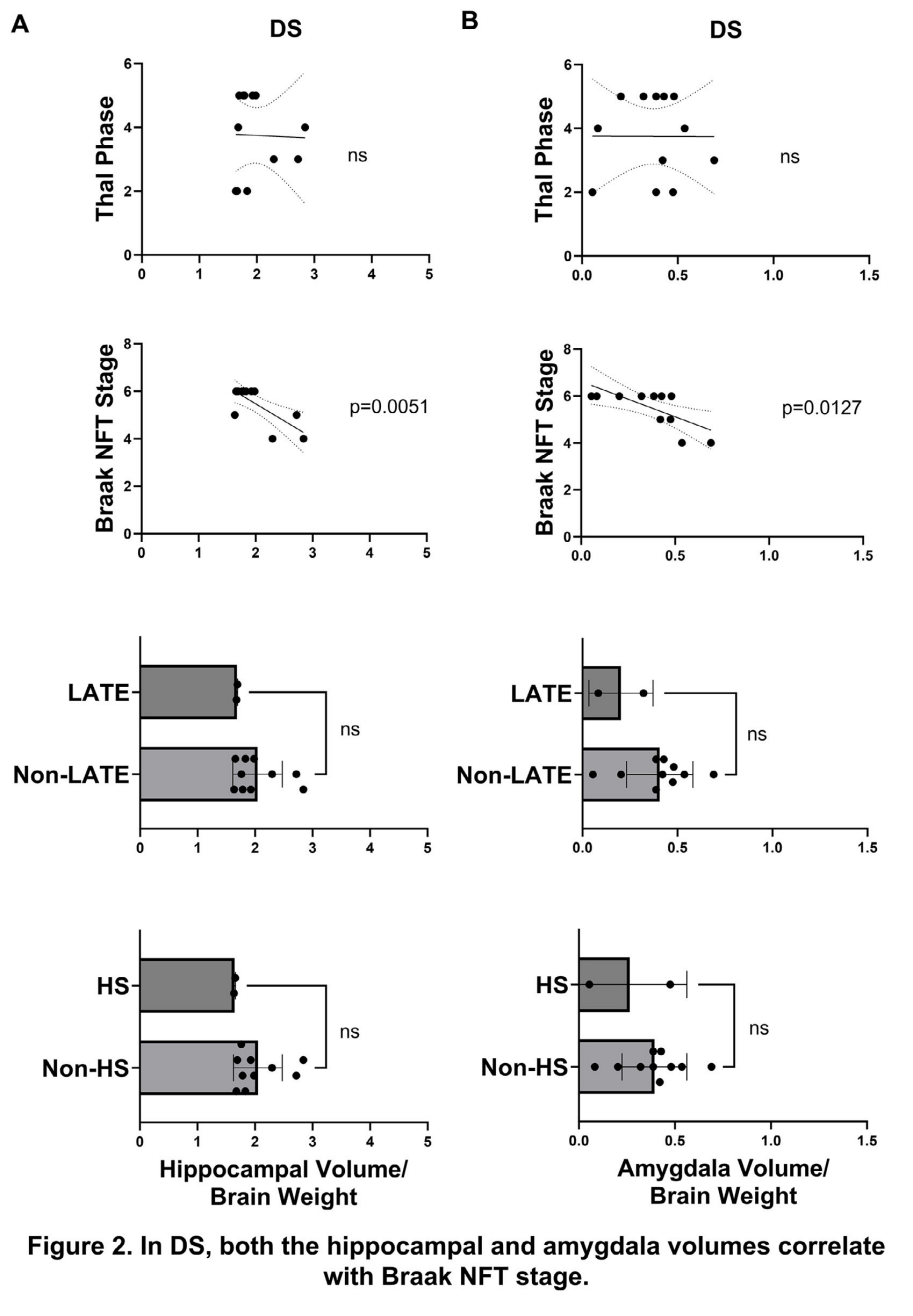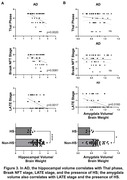# Correlating hippocampal and amygdala volumes with neuropathological burden in neurodegenerative diseases using 7T postmortem MRI

**DOI:** 10.1002/alz.083741

**Published:** 2025-01-09

**Authors:** Jr‐Jiun Liou, Jinghang Li, Jacob Berardinelli, Hecheng Jin, Tales Santini, Jaehoon Noh, Nadim Farhat, Howard J Aizenstein, William Yong, Elizabeth Head, Milos D Ikonomovic, Tamer S Ibrahim, Julia Kofler

**Affiliations:** ^1^ University of Pittsburgh, Pittsburgh, PA USA; ^2^ University of California, Irvine, Irvine, CA USA

## Abstract

**Background:**

High‐resolution and high‐contrast postmortem MRI can unveil structural abnormalities overlooked by antemortem MRI, making it well‐suited for targeted pathology assessments. In community cohorts, limbic‐predominant age‐related TDP‐43 encephalopathy (LATE) pathology is linked with smaller amygdala [Makkinejad 2019 Neurobiol Aging] and hippocampal volumes [Josephs 2015 Ann Neurol]. Whether a similar association exists in Down syndrome (DS) remains unexplored. This study investigates the potential correlation of amygdala and hippocampal atrophy with AD and LATE pathology in both DS and AD.

**Methods:**

7T postmortem ex vivo MRI scans (n=67, comprising 13 DS and 54 AD) and a subset of antemortem MRI scans (n=17) were obtained. Manual segmentation of the hippocampus and amygdala was performed. The resulting volumes, normalized to fresh brain weight and adjusted for age, sex, and ApoE4, were correlated with pathological findings, including Thal phase, Braak stage, LATE stage, and the presence of hippocampal sclerosis (HS).

**Results:**

Postmortem‐antemortem volume correlation was significant for the hippocampus (p=0.0066) but not the amygdala. Compared to AD, individuals with DS had significantly lower hippocampal (p=0.0004) and amygdala (p=0.0002) volumes. In the DS group, lower hippocampal (p=0.0051) and amygdala (p=0.0127) volumes correlated with more severe Braak stage, with no significant correlations with Thal phase. In two DS cases that exhibited LATE or HS, trends toward smaller hippocampal volume were observed. In AD, lower hippocampal volume correlated with more severe Thal phase (p=0.0020), Braak stage (p=0.0081), LATE stage (p=0.0017), and the presence of HS (p<0.0001), whereas lower amygdala volume correlated with more severe LATE stage (p=0.0160) and HS (p=0.0139) but not with Thal phase or Braak stage.

**Conclusions:**

7T postmortem MRI protocol produced good alignment of postmortem volumes with antemortem findings and revealed correlations between volume measures and neuropathological burden. In AD, hippocampal volume is influenced by both AD and LATE pathologies, whereas amygdala volume appears to be influenced primarily by LATE. In DS, hippocampal volume is smaller than in AD and primarily influenced by tau pathology. Future investigation will also explore Lewy body pathology and its potential association with hippocampal and amygdala volume changes in DS and AD.